# Expression of Mitochondrial Cytochrome C Oxidase Chaperone Gene (*COX20*) Improves Tolerance to Weak Acid and Oxidative Stress during Yeast Fermentation

**DOI:** 10.1371/journal.pone.0139129

**Published:** 2015-10-01

**Authors:** Vinod Kumar, Andrew J. Hart, Ethiraju R. Keerthiraju, Paul R. Waldron, Gregory A. Tucker, Darren Greetham

**Affiliations:** University of Nottingham, School of Biosciences, Sutton Bonington Campus, Loughborough, LE12 5RD, United Kingdom; National Renewable Energy Lab, UNITED STATES

## Abstract

**Introduction:**

*Saccharomyces cerevisiae* is the micro-organism of choice for the conversion of fermentable sugars released by the pre-treatment of lignocellulosic material into bioethanol. Pre-treatment of lignocellulosic material releases acetic acid and previous work identified a cytochrome oxidase chaperone gene (*COX20*) which was significantly up-regulated in yeast cells in the presence of acetic acid.

**Results:**

A Δ*cox20* strain was sensitive to the presence of acetic acid compared with the background strain. Overexpressing *COX20* using a tetracycline-regulatable expression vector system in a Δ*cox20* strain, resulted in tolerance to the presence of acetic acid and tolerance could be ablated with addition of tetracycline. Assays also revealed that overexpression improved tolerance to the presence of hydrogen peroxide-induced oxidative stress.

**Conclusion:**

This is a study which has utilised tetracycline-regulated protein expression in a fermentation system, which was characterised by improved (or enhanced) tolerance to acetic acid and oxidative stress.

## Introduction

Fossil-based hydrocarbon fuels for generating energy, such as coal and crude oil, are finite resources and at the present rate of human consumption are predicted to be completely depleted by 2050 [1, 2]. An alternative, renewable source of energy is lignocellulosic residue from agricultural, forestry, municipal or industrial processes [3]. Sugars can be released from the lignocellulosic feed stocks using industrial pre-treatment processes, followed by enzymatic digestion and then converted to transportation biofuels, such as bioethanol or biobutanol by microbial fermentation [4].

*Saccharomyces cerevisiae* is currently used for the production of bioethanol, first generation bioethanol production has involved the conversion of hexose sugars derived from sucrose present in crops such as sugar cane in Brazil and from starch in crops such as maize in the USA [5]. Use of lignocellulosic feed stocks for biofuel production is more challenging, in order to increase fermentation efficiency, the problem of pre-treatment generated inhibitor compounds, and fermentation stresses, also have to be addressed. Pre-treatment of lignocellulose to release constituent sugars results in the release of aromatic and acidic compounds such as acetic acid, formic acid, furfural, hydroxy-methyl furfural (HMF), and vanillin [6] that are detrimental to the growth of *S*. *cerevisiae*. In addition, fermentations carried out within bioreactors generates further problems, such as osmotic stress due to high sugar levels, elevated heat and increasing ethanol concentrations [7–9]. Thus, resistance to all these fermentation stresses are desirable phenotypic attributes for improved bioethanol productivity.

Using a systems biology approach and a phenotypic microarray assay we previously identified quantitative trait loci (QTLs) associated with response to weak acids [10], recipricol hemizygosity analysis of genes within the loci highlighted the potential role of *COX20* in weak acid response [10]. Cytochrome C oxidase activity has been associated with programmed cell death (PCD) in yeast [11], where a loss of function along with addition of acetic acid has been shown to induce PCD [12]. Cytochrome C release has been shown to involve the ATP/ADP carrier as a component of the mitochondrial outer membrane [11] and has been shown to be released in response to reactive oxygen species (ROS) [13]. Yeast strains with altered cytochrome C oxidase activity maybe more tolerant to the inducement of PCD by acetic acid, the importance of cytochrome C oxidase has been reported in work on improving acetate tolerance in *E*. *coli* [14]. *COX20* encodes for a chaperone that facilitates proteolytic processing of the mitochondrial gene product Cox2p and its assembly into the mitochondrial inner membrane cytochrome C oxidase complex [15]. In the work reported here, *COX20* was expressed using tetracycline-regulatable vectors [16] in a Δ*cox20* strain and response to acetic acid was measured using phenotypic microarrays and during fermentation. Incomplete assembly or mis-functioning cytochrome C oxidases have been associated with elevated levels of oxidative stress in the yeast cell [17] so the effect of oxidative stress on COX20 was also investigated.

## Material And Methods

### Yeast strains and growth conditions

Yeast strains employed in this work derive from *S*. *cerevisiae* BY4741 (w) (Table 1). All strains were grown in YPD [1% (w/v) yeast extract (Oxoid); 2% (w/v) Bacto-peptone (Oxoid); 2% (w/v) glucose]. Strains were deleted for tryptophan biosynthesis (*trp1*: URA3) using pAG60 (Euroscarf, Frankfurt, Germany) with a URA3 selectable marker using primers (Table 2). A *cox20* null mutant was obtained from Euroscarf (Frankfurt, Germany) and *TRP1* was also deleted from this strain as above for wild-type (BY4741).

**Table 1:** Strains used in this study, all strains are derived from BY4741 (MATa *his3*Δ0 *leu2*Δ0 *met15*Δ0 *ura3*Δ0), with the additional *TRP1* gene knocked out to allow for selection with pCM plasmids. * Integrative plasmids were constructed as indicated in material and methods.

Strains used in this study	Integrative plasmid*
BY4741-*trp1* (MATa *his3*Δ0 *leu2*Δ0 *met15*Δ0 *ura3*Δ0 *trp1*Δ0)	-
BY4741-*trp1*	pCM161
BY4741-*trp1*	pCM173
BY4741-*trp1*-*cox20*	-
BY4741-*trp1*-*cox20*	pCM161
BY4741-*trp1*-*cox20*	pCM161(*COX20*)
BY4741-*trp1*-*cox20*	pCM173
BY4741-*trp1*-*cox20*	pCM173(*COX20*)

**Table 2:** Primers used in this study for the knockout of *TRP1* and insertion of pCM vectors into yeast strains.

Primers	Sequence
*TRP1* knockout forward	CGCCAGATGGCAGTAGTGGAAGATATTCTTTATTGAAAAATAGCTTGTCAATGACAGTCAACACTAAGACCTATA
*TRP1* knockout reverse	TTTTATGCTTGCTTTTCAAAAGGCCTGCAGGCAAGTGCACAAACAATACTTTATAATTGGCCAGTCTTTTTC
pCM161 forward	GTCGAACATGCGTTGGTGGCCGTGGTC
pCM161 reverse	GATATCTCACCAGAACTTGTACCATT
pCM173 forward	GGATCCATGCGTTGGTGGCCGTGGTC
pCM173 reverse	ATCGATTCACCAGAACTTGTACCATT

### Plasmid construction

Plasmids pCM161 and pCM173 are centromeric yeast plasmids, marker *TRP1*, tetracycline repressed expression of lacZ. These plasmids are under the control of the tetO2 and tetO7 promoters, respectively. To construct them, a PCR product encoding for *COX20* with relevant restriction enzyme sites appropriate for ligation into the vectors was prepared. For cloning into pCM161 *Sal*I and *EcoRV* restriction enzymes (NEB) were used and the relevant digest site added to the forward and reverse primers (Table 2). For cloning into pCM173 *ClaI* and *BamHI* (NEB) restriction enzymes were used and added to the relevant primers.

### PCR of *COX20* from genomic DNA

*S*. *cerevisiae* wild-type (BY4741) was grown to stationary phase, cells harvested and broken with glass beads using a MagNalyser (Roche, Burges Hill, UK) bead beater (7000 rpm) for 30 seconds at 4°C, before incubating on ice for 15 min to precipitate proteins. Cell debris and proteins were harvested by centrifugation for 15 min (17,000 x g at 4°C). The cell-free supernatant was used for the extraction of total DNA using an isolation kit (Pierce, US, Catalogue no: 78870). PCR was performed using primers (Table 2). PCR products using above primers were digested and linearized, digested plasmids with appropriate restriction enzymes were ligated using the Quick ligation mix (NEB, US, Catalogue no: M2200S) and the ligation mixture used in a standard lithium acetate transformation [18] and plated out on appropriate selection plates.

### β-galatosidase activity

β-galactosidase activity was determined using a β-galactosidase reporter gene activity detection kit (Sigma-Aldrich, US). 100 mL yeast cells containing a LacZ gene were grown to mid-exponential phase (OD600 nm = 0.5) in YPD at 30°C, 180 rpm in an orbital shaker, in aerobic conditions. Cell pellets were obtained by centrifugation at 13200 x *g*, 4°C for 4 min and resuspended in 200 μL lysis buffer, addition of 0.25 g of Biospec Products, Inc 0.5 mm diameter soda lime glass chilled beads (catalogue number 11079105). Cell pellets were broken by vigorous shaking/vortexing via a MagNa lyser (Roche, Applied Science, UK) for 1 min and repeated five times at a speed of 7,000 rpm. During the breaking temperature was kept as low as practicable. The cell lysate was centrifuged at 17, 000 g for 1 min at 4°C and the supernatant retained for the β-galactosidase assay. Assay proceeded as described in the kit from this point onwards. Data was representative of triplicate experiments.

### SDS-PAGE analysis

Yeast cells were grown in 100 mL YPD at 30°C at 180 rpm in an orbital shaker under aerobic conditions until an OD_600_ of 1 was reached. Cell pellets were obtained by centrifugation at 13200 x *g* at 4°C for 4 min and were re-suspended in 160 μL of polymerase chain reaction (PCR) grade water containing Fisher Scientific yeast oriented protease inhibitor cocktail (catalogue number BPE9711-13) (1:10 protease inhibitor to PCR grade water). To the pellet was added 0.25 g of 0.5 mm diameter soda lime glass chilled beads (Biospec Products, Inc). The cells were then broken using a MagNA lyser (Roche Applied Science, UK) and the cells were subjected to vigorous shaking/vortexing via the MagNa lyser for 1 min and repeated five times at a speed of 7,000 rpm while temperature was kept as low as practicable.

Precast gels (Bio-Rad Laboratories, UK) were used for assessment of protein extracts via SDS-PAGE experiments. 10 μL of 1M DTT and Laemmli sample loading buffer composed of 4% (w/v) SDS, 120 mM of 1M Tris-HCl (pH6.8) and bromophenol blue to a final concentration of 0.02% (w/v) [19] was added to 10 μL of protein sample and heated at 90°C for 5 min on a heating block. Samples were then loaded in wells alongside molecular weight pre-stained broad range molecular weight marker (Bio Rad Laboratories, UK). Gels were stained in Coomassie R250 for visualisation.

### Quantitative PCR analysis

Strains (wild-type (BY4741), Δ*cox20*, and Δ*cox20* (containing plasmids)) were grown to mid-logarithmic stage of growth in YPD at 30°C, stressed by the addition of 25 mM acetic acid and rotated at 150 rpm. Cells were broken with glass beads using a MagNa lyser (Roche, Burges Hill, UK) bead beater for 30 seconds at 4°C at 7000 rpm, before incubating on ice for 15 min to precipitate proteins. Cell debris and proteins were harvested by centrifugation for 15 min (17,000 x g at 4°C). The cell-free supernatant was used for the extraction of total RNA using an isolation kit from Qiagen (Hilden, Germany) (typical RNA levels were 1–3 mg/mL, with no genomic DNA present) and cDNA prepared using a first strand cDNA synthesis kit (GE Healthcare, Bucks, UK). Transcriptional levels were determined by qPCR using the following conditions: 0.5 ng/μL cDNA, 6.25 μM forward primer, 6.25 μM reverse primer, 5 μL of 2 x SYBR Green master mix (Applied Bio Systems) and made up to 20 μL using molecular grade water. All data was compared against *ACT1* which encodes for actin a structural protein in yeast as an internal normaliser and expression data from genes within the relevant loci were presented as fold-change in comparison to *ACT1* transcript levels in control and stress conditions.

### Phenotypic microarray analysis

For phenotypic microarray (PM) analysis, medium was prepared as described previously [20]. Stock solutions of acetic acid (1M) and hydrogen peroxide (8.8 M) were prepared using RO sterile water. Cell suspensions for the inoculums were prepared by mixing 125 μL of the above cells with IFY-0 buffer ™ (Biolog) and the final volume adjusted to 3 mL using RO sterile distilled water. 90 μL of the above mix was inoculated into each well in a Biolog 96-well plate. Anaerobic conditions were created using Oxygen absorbing packs (Mitsubishi AnaeroPak™System address) with an anaerobic indicator (Oxoid, Basingstoke, UK) and the plates were placed inside PM gas bags (Biolog). The plates were then placed in the OmniLog reader and incubated for 50 h at 30°C.

The OmniLog reader photographed the PM plates at 15 min intervals, and converted the pixel density in each well to a signal value reflecting cell growth and dye conversion. Dye reduction which reflects metabolic activity of cells has been defined here as the redox signal intensity. After completion of the run, the signal data was compiled and exported from the Biolog software using Microsoft^®^ Excel. In all cases, a minimum of three replicate PM assay runs were conducted, and the mean signal values are presented.

### Measurement of yeast growth

Yeast growth under identical growth conditions as for PM assays was monitored for 50 hrs with a reading every 15 minutes using a Tecan (Mannedorf, Switzerland) Infinite M200 Pro plate reader, at 30°C for 50 hrs. The assay was performed in triplicate and an average reading was plotted.

### Sensitivity to acetic acid or hydrogen peroxide-induced oxidative stress

Sensitivity to acetic acid or hydrogen peroxide-induced oxidative stress was determined by growing cells to stationary phase in YPD at 30°C. Optical density (OD_600_) was measured and adjusted to an OD_600_ of 1.0 corresponding to 10^7^ cells/mL. Cells were then serially ten-fold diluted and 5 μL was spotted onto YPD agar plates containing acetic acid (0–100 mM) or hydrogen peroxide (0–1 mM) as appropriate.

### Confirmation of phenotypic microarray results using mini fermentation vessels

Fermentations were conducted in 180 mL mini-fermentation vessels (FV). Cryopreserved yeast colonies were streaked onto YPD plates and incubated at 30°C for 48 hrs. Colonies of yeast strains were used to inoculate 20 mL of YPD broth and incubated in an orbital shaker at 30°C for 24 hrs. These were then transferred to 200 mL of YPD and grown for 48 hrs in a 500 mL conical flask shaking at 30°C. Cells were harvested and washed three times with sterile RO water and then re-suspended in 5 mL of sterile water. For control conditions, 1.5 × 10^7^ cells mL^−1^ were inoculated in 99.6 mL of SD-trp medium (4% glucose, 2% YNB-trp) with 0.4 mL sterile distilled water. For stress conditions, 1.5 × 10^7^ cells mL^−1^ were incubated in 99.6 mL of medium containing 4% glucose, 2% YNB-trp with 75 mM acetic acid, Volumes of media were adjusted to account for the addition of the inhibitory compounds (~400 μL) to ensure that all fermentations began with the same carbon load.

Anaerobic conditions were prepared using a sealed butyl plug (Fisher, Loughborough, UK) and aluminium caps (Fisher Scientific). A hypodermic needle attached with a Bunsen valve was purged through rubber septum to facilitate the release of CO_2._ All experiments were performed in triplicate and weight loss was measured at each time point. Mini-fermentations were conducted at 30°C, with orbital shaking at 200 rpm. Fermentations looking at pH had SD-trp media adjusted to pH 2.2, 1.7 or 1.2 (analogous to the pH of the media in the presence of 25, 50 or 75 mM acetic acid respectively) with phosphoric acid.

### Preparing acid pre-treated hydrolysates from wheat for phenotypic microarray assays

1 g of air-dried wheat straw, that had been knife-milled to a 2 mm particle size, was added to 10 mL of 1% sulphuric acid and heated at 121°C for 30 min in an autoclave (Priorclave autoclave Tactrol 2). The samples were then centrifuged for 5 min at 4472g. Supernatants were removed and discarded. The solid residue was washed four times with 40 mL of de-mineralised water. Following the final rinse the samples were passed through a filter paper (Whatman, GF/A, 110mm) and the solid residue collected and allowed to air dry for 48 h. Dry samples were then placed into labelled Falcon tubes and frozen until needed.

Enzymatic hydrolysis was carried out using a slight modification to the method described by the National Research Energy Laboratory (NREL) (Selig et al 2008). A stock of cellulase enzyme was prepared by dissolving 200 mg of cellulase (Sigma-Aldrich) from *Trichoderma reesei*, ATCC 20921, lot # 31M1283V into 20 mL of 50 mM sodium citrate buffer (pH 4.8). 200 mg of air dried biomass was placed into a numbered 50 mL Falcon tube and 36 mL of 50 mM sodium citrate buffer (pH 4.8) was added.

4 mL of the stock cellulase enzyme was then added to the numbered 50 mL Falcon tube to make a 0.005 wt/wt dispersion at 40 FPU per g/ biomass. The samples were then placed horizontally into a shaking incubator (Sarorius Stedim biotech certomat BS1) at 50°C and 150 rpm. After 15 min the tubes were removed and 0.1 mL of the sample was then placed into a labelled 15 mL Falcon tube and 9.9 mL of 10 mM NaOH added. pH post digestion was around pH 2, so pH was adjusted to a starting pH of pH 5 using 5 M NaOH.

These hydrolysates contained very low glucose concentrations, so 9 μL of 80% glucose was added to each well giving a 6% starting percentage for phenotypic microarray assays along with 0.2 μL dye D and 90 μL cells as described above.

### Detection of glucose and ethanol from FV experiments via HPLC

Glucose and ethanol were quantified by HPLC. The HPLC system included a Jasco AS-2055 Intelligent auto sampler (Jasco, Tokyo, Japan) and a Jasco PU-1580 Intelligent pump (Jasco). The chromatographic separation was performed on a Rezex ROA H^+^ organic acid column, 5 μm, 7.8mm x 300 mm, (Phenomenex, Macclesfield, UK) at ambient temperature. The mobile phase was 0.005N H_2_SO_4_ with a flow rate of 0.5 mL/min. For detection a Jasco RI-2031 Intelligent refractive index detector (Jasco) was employed. Data acquisition was via the Azur software (version 4.6.0.0, Datalys, St Martin D’heres, France) and concentrations were determined by peak area comparison with injections of authentic standards. The injected volume was 10 μL and analysis was completed in 40 minutes. All chemicals used were analytical grade (>95% purity, Sigma-Aldrich, UK).

### Statistical analysis

Data derived from phenotypic microarrays was analysed for analysis of variance (ANOVA) using ezANOVA (http://www.cabiatl.com/mricro/ezanova), a free–for-use online statistical program with statistical significance signified by use of *, * = data significant at the 0.05% confidence interval. ** = data significant at the 0.01% confidence interval and *** data significantly different at the 0.001% confidence interval.

## Results

### Confirmation of tetracycline-regulated expression of *COX20*

**Fig 1:**
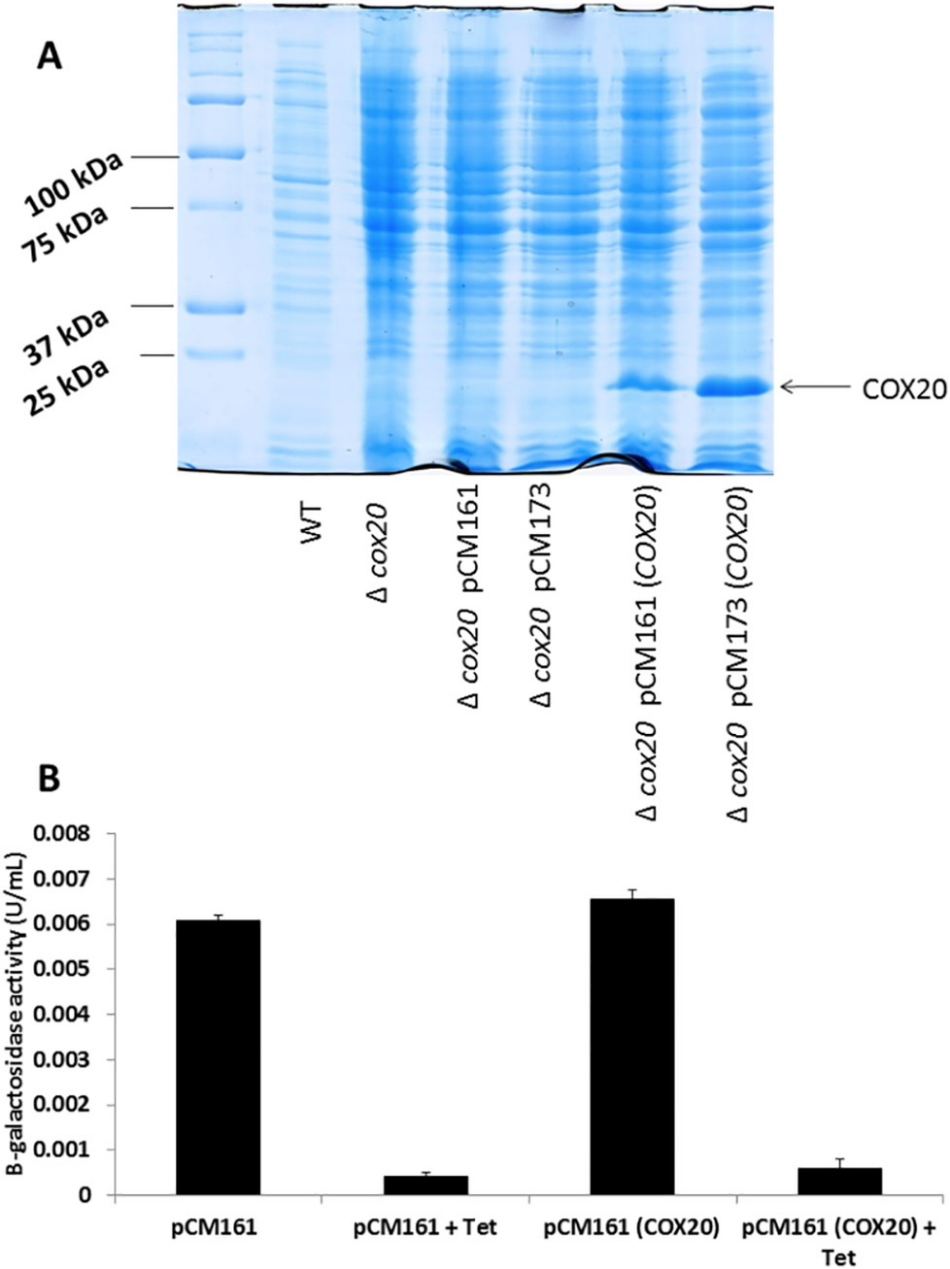
(A) 10% SDS-PAGE gel of protein samples from wild-type (BY4741), Δ*cox20*, Δ*cox20* pCM161, Δ*cox20* pCM173, Δ*cox20* pCM161(*COX20*), Δ*cox20* pCM173(*COX20*). (B) β-galactosidase activity (U/ mL) for Δ*cox20* (pCM161), Δ*cox20* (pCM161) + 1 μg/mL tetracycline, Δ*cox20* (pCM161(*COX20*)), Δ*cox20* (pCM161(*COX20*)) + 1 μg/mL tetracycline. Results presented are a representative of triplicate values (Mean *+*/- SD n = 3).

Centromeric plasmids containing *COX20* under the regulation of tetO2 (pCM161) or tetO7 (pCM173) promoters were constructed; these were then used to transform a Δ*cox20* yeast strain. The Δ*cox20* strain was also transformed with empty vector as a further control. The wild type and all the transformed strains were assessed for expression of COX20 on a SDS-PAGE gel (Fig 1A). COX20 has a predicted molecular weight of 23 KDa [15]. There was no detectable expression in the wild type, Δ*cox20* or either of the empty vector controls (Fig 1A), a clear band was evident in both over-expression lines, protein concentrations being higher in the strain with the tetO7 regulated promoter (pCM173(*COX20*)) than the strain with a tet02 regulated promoter (pCM161(*COX20*)) (Fig 1A). Expression from these two promoters is suppressed by the addition of tetracycline (1 μg/mL) [16], and this was demonstrated in this instance as the addition of tetracycline revealed that there was no detectable protein expression (data not shown). Expression of β-galactosidase is an additional method to test the efficiency of tetracycline repression and activity was detected for a Δ*cox20* strain carrying either a pCM161 vector or a pCM161 vector containing *COX20*. Addition of tetracycline suppressed β-galactosidase activity in each case (Fig 1B).

### Expression of *COX20* rescues Δ*cox20* sensitivity to weak acids

**Fig 2:**
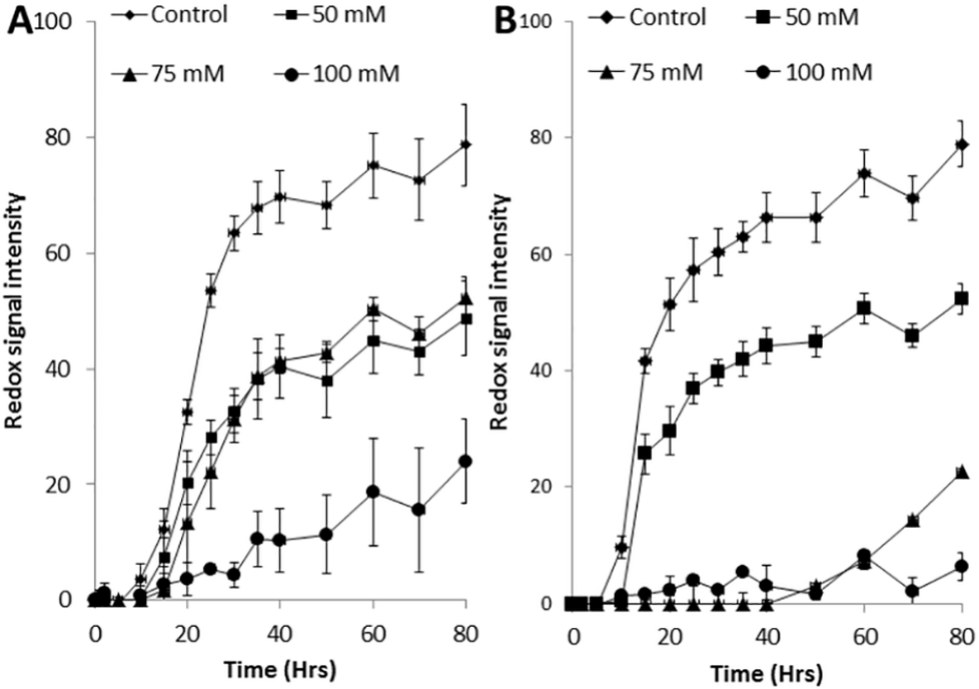
Metabolic activity of WT and Δ*cox20* under 0–100 mM acetic acid, (A) WT and (B) Δ*cox20*. Results presented are a representative of triplicate values (Mean *+*/- SD n = 3).

**Fig 3:**
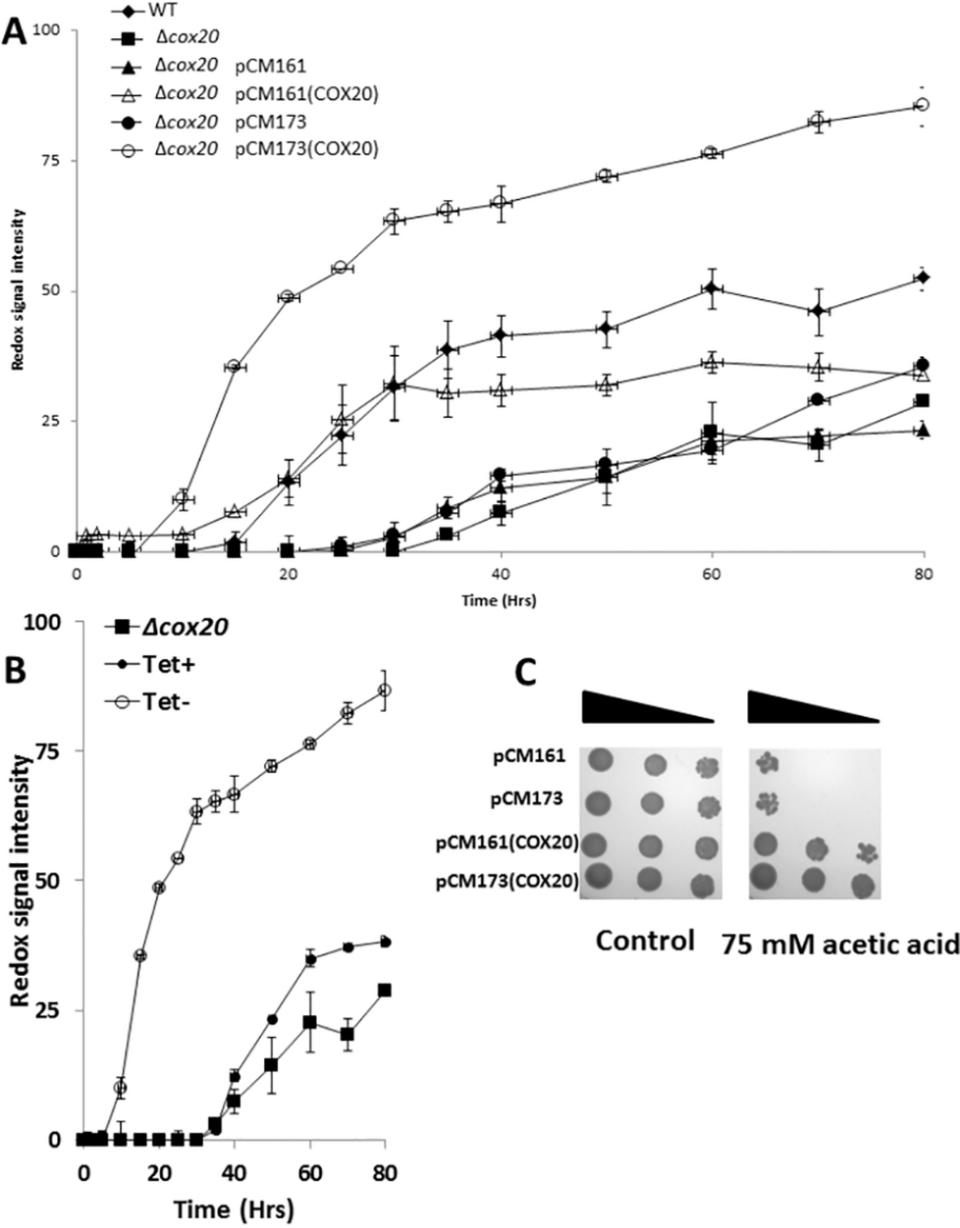
(A) Metabolic activity of WT, Δ*cox20*, Δ*cox20* pCM161, Δ*cox20* pCM173, Δ*cox20* pCM161(*COX20*), Δ*cox20* pCM173(*COX20*) grown under 75 mM acetic acid, (B) Metabolic activity of Δ*cox20 and* Δ*cox20* pCM173(*COX20*) with and without tetracycline, under 75 mM acetic acid (C) Spot plate analysis of Δ*cox20* pCM161, Δ*cox20* pCM173, Δ*cox20* pCM161(*COX20*), Δ*cox20* pCM173(*COX20*) in the presence of control and 75 mM acetic acid. Results presented are a representative of triplicate values (Mean *+*/- SD n = 3).

The metabolic activity of wild type (BY4741) and the Δ*cox20* strain during incubation in the presence of acetic acid (0–100 mM) was determined by use of a phenotypic microarray (Fig 2A and 2B). The two strains demonstrated similar metabolic activity under control (no acetic acid) conditions but the Δ*cox20* strain was characterised by an increase in acetic acid sensitivity when compared with wild-type in particular at higher concentrations. The impact of the overexpression of *COX20* on tolerance to acetic acid was then assessed, Δ*cox20* strains which had been transformed with plasmids (pCM161 or pCM173) containing *COX20* under a tetracycline-regulatable promoter were assessed for acetic acid tolerance in comparison with their empty vector controls (Fig 3A). Presence of empty vectors (pCM161 or pCM173) had no discernible effect on tolerance to 75 mM acetic acid when compared to the Δ*cox20* background (Fig 3A). However, Δ*cox20* strains containing the overexpression vectors pCM161(*COX20*) or pCM173(*COX20*) displayed increased tolerance to 75 mM acetic acid as compared to their corresponding empty vector controls (Fig 3A). The effect of pCM173(*COX20*) being particularly effective at increasing metabolic activity, compatible with the fact that this construct employs a stronger promoter. Protein expression using a pCM173 vector has been shown to be higher when compared with expression level using a pCM161 vector [16]. The metabolic activity of the wild type yeast under these conditions was intermediate between the Δ*cox20* and pCM173 (*COX20*) strains (Fig 3A). Assays with Δ*cox20* strains expressing *COX20* in a pCM161 plasmid appeared to recover the phenotype when compared with wild-type for the first 30 hours of the assay; after this time point there was no further increase metabolic output when compared with wild-type. For this reason all further studies were undertaken using the pCM173(*COX20*) strain. Tolerance to acetic acid was further confirmed in assays using 100 mM acetic acid in which a strain with an empty vector was sensitive whereas a strain overexpressing COX20 was resistant (S1A Fig).

Addition of tetracycline (1 μg/mL) reverted the Δ*cox20* strain with pCM173(*COX20*) back to be susceptibile to inhibition by acetic acid when compared with absence of tetracycline (Fig 3B). It was also observed that a Δ*cox20* strain overexpressing *COX20* displayed tolerance to 20 mM formic acid when compared with a Δ*cox20* strain (S1B Fig). Assays looking at tolerance to other stresses in strains overexpressing *COX20* revealed that there was no difference in strains overexpressing *COX20* in the presence of increased osmotic stress, temperature, furanic, or phenolic stress (data not shown).

Increased tolerance to acetic acid in overexpression strains was further confirmed by spotting onto SD medium lacking tryptophan to select for the plasmid. Overexpression of *COX20* was found to significantly increase resistance to acetic acid in a Δ*cox20* strain (Fig 3C).

### Presence of *COX20* improves growth in the presence of acetic acid

**Fig 4:**
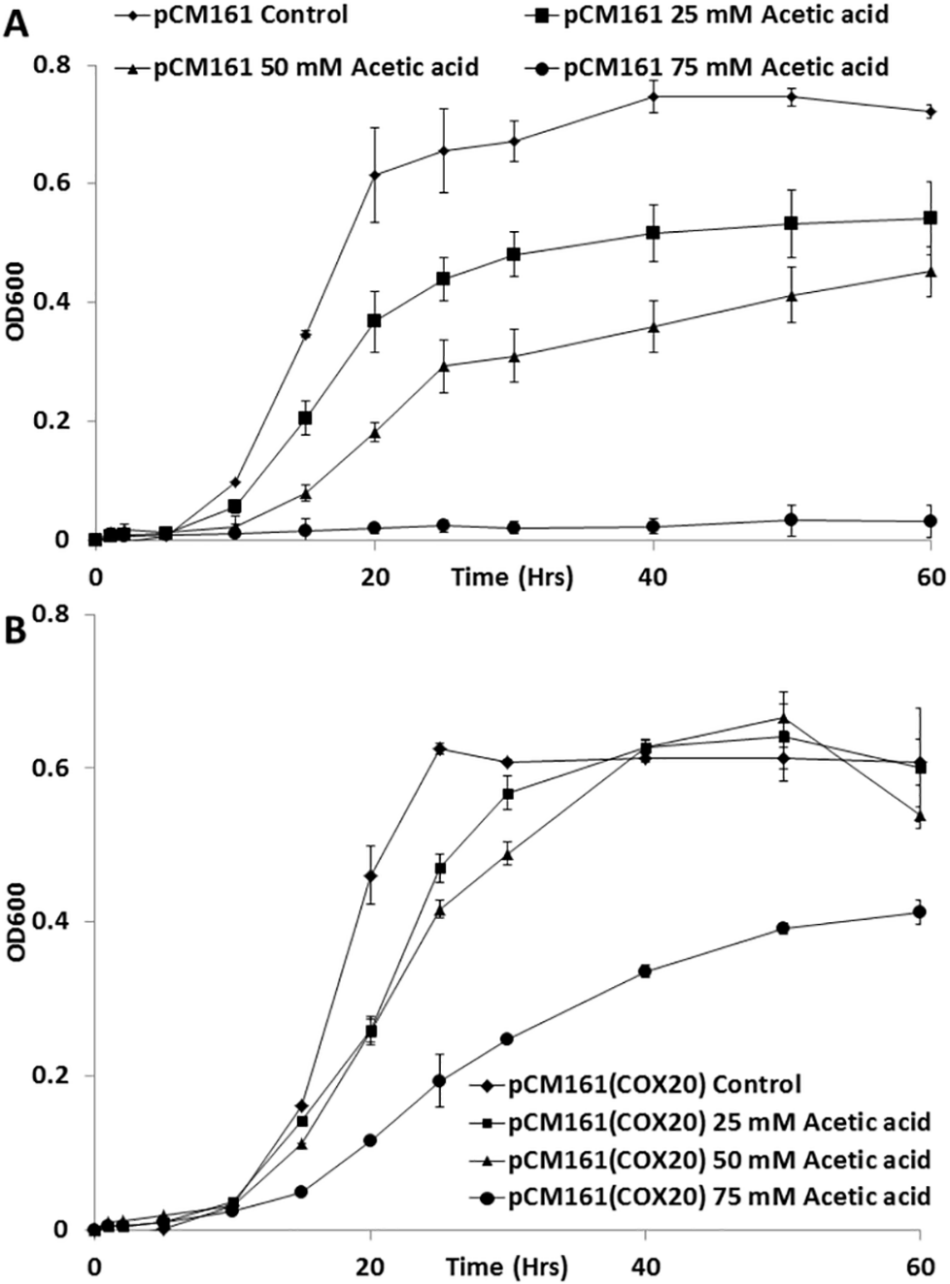
Growth rates for Δ*cox20* pCM173 and Δ*cox20* pCM173(COX20) under control conditions (4% glucose) and 25–75 mM acetic acid (A) Δ*cox20* pCM173 and (B) Δ*cox20* pCM173(COX20). Results presented are a representative of triplicate values (Mean *+*/- SD n = 3).

The growth of yeast strains overexpressing *COX20* was determined in the presence of acetic acid and compared with the empty vector control. Yeast strains containing pCM173 or pCM173(*COX20*) had similar growth curves under control conditions (Fig 4A and 4B), however; addition of 25–75 mM acetic acid slowed growth of a yeast strain containing an empty pCM173 vector when compared with a strain containing a pCM173(*COX20*) vector (Fig 4A and 4B). An assessment of half maximal inhibitory concentration (IC_50_) for acetic acid on growth revealed that Δ*cox20* and a Δ*cox20* (pCM173) strain had IC_50_s of 76 ± 0.12 and 77.5 ± 0.26 mM respectively, wild-type (BY4741) had an IC_50_ value of 83 ± 1.15 mM and a Δ*cox20* (pCM173) had an IC_50_ value of 98.5 ± 0.9 mM respectively.

### Confirmation of phenotypic microarray strain assessments using mini-fermentation analysis

Performance of yeast strains in fermentations under acetic acid stress was assessed. The Δ*cox20* strain carrying either an empty vector (pCM173) or overexpressing *COX20* (pCM173(*COX20*)) were used in fermentations carried out under control (no acetic acid) or in the presence of 75 mM acetic acid and fermentation efficiency monitored gravimetrically. SD-trp media was used to maintain selection criteria for the plasmid (S2 Fig). Under control conditions, there was no discernible difference in fermentation profiles between the two strains (p = 0.8765) (S2 Fig). In contrast, the pCM173(*COX20*) strain was tolerant to the presence of acetic acid when compared with an empty vector control strain (p = 0.0006) (S2 Fig). When fermentation profiles for the pCM173(*COX20*) strain were compared under control and acetic acid stress conditions, there were no significant differences (p = 0.427) (S2 Fig).

**Fig 5:**
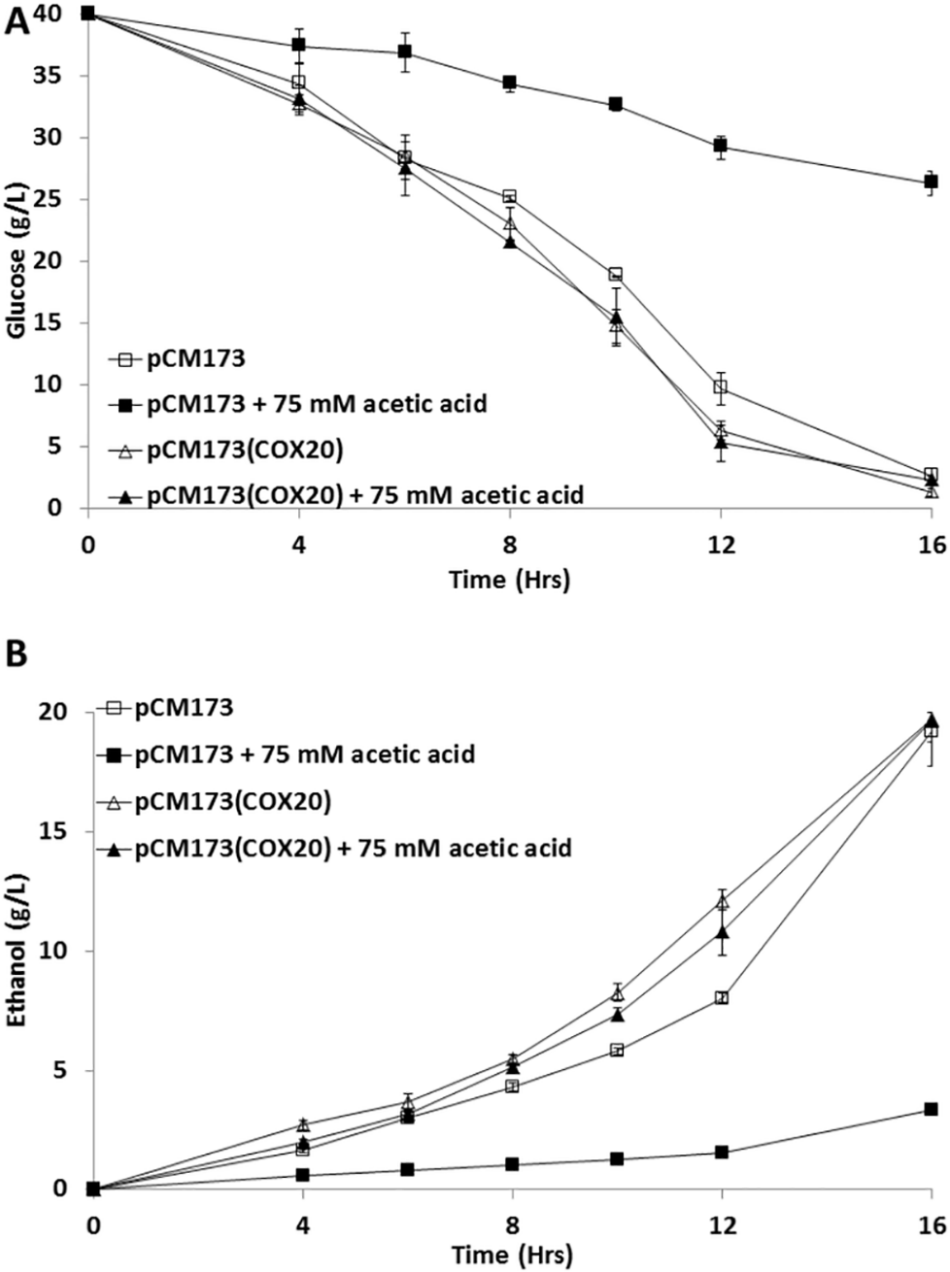
(A) Glucose utilisation (g/L) during a fermentation for a Δ*cox20* (pCM173) and Δ*cox20* (pCM173(*COX20*)) under control and in the presence of 75 mM acetic acid, (B) ethanol production (g/L) during a fermentation for a Δ*cox20* (pCM173) and Δ*cox20* (pCM173(COX20)) under control and in the presence of 75 mM acetic acid. Results presented are a representative of triplicate values (Mean *+*/- SD n = 3).

The fermentation outputs of the two strains under control and 75 mM acetic acid stress were also assessed in terms of glucose utilisation and ethanol production (Fig 5). Under control conditions, it was observed that there was no difference in glucose utilisation or ethanol production between the strains with either the empty vector or pCM173(*COX20*) (p = 0.861) (Fig 5A and 5B). Addition of 75 mM acetic acid significantly reduced glucose utilisation and ethanol production in the empty vector control strain as compared to the strain overexpressing *COX20* (p = 0.0168) (Fig 5A and 5B). This data were used to assess the efficiency of the conversion of glucose into ethanol. Under control conditions, the empty vector Δ*cox20* control strain had 0.48 ± 0.011 g ethanol/g glucose conversion efficiency, and the pCM173(*COX20*) strain had an efficiency of 0.49 ± 0.007 ethanol/g glucose conversion after 12 hours. In the presence of 75 mM acetic acid, the Δ*cox20* control strain had a 0.08 ± 0.008 ethanol/g glucose conversion whereas the pCM173(*COX20*) strain had a 0.49 ± 0.045 ethanol/g glucose conversion. The theoretical maxima is 0.511 g ethanol per g of glucose consumed [21], therefore the pCM173(*COX20*) strain is converting glucose into ethanol at near theoretical maximum in the presence of 75 mM acetic acid. The impact of pH was measured on pCM173 and pCM173(*COX20*), selective media was adjusted to a pH analogous to the addition of 25, 50 or 75 mM acetic acid using phosphoric acid and fermentation rates monitored. Presence of acetic acid in a fermentation using either empty vector (pCM173) or (pCM173(COX20)) was unchanged under control conditions or in the presence of acetic acid (S3 Fig). Results revealed that presence of COX20 had no discernible effect on tolerance to pH when compared with the empty vector (pCM173) control (data not shown).

### Overexpression of *COX20* improves yeast tolerance to hydrogen peroxide-induced oxidative stress

**Fig 6:**
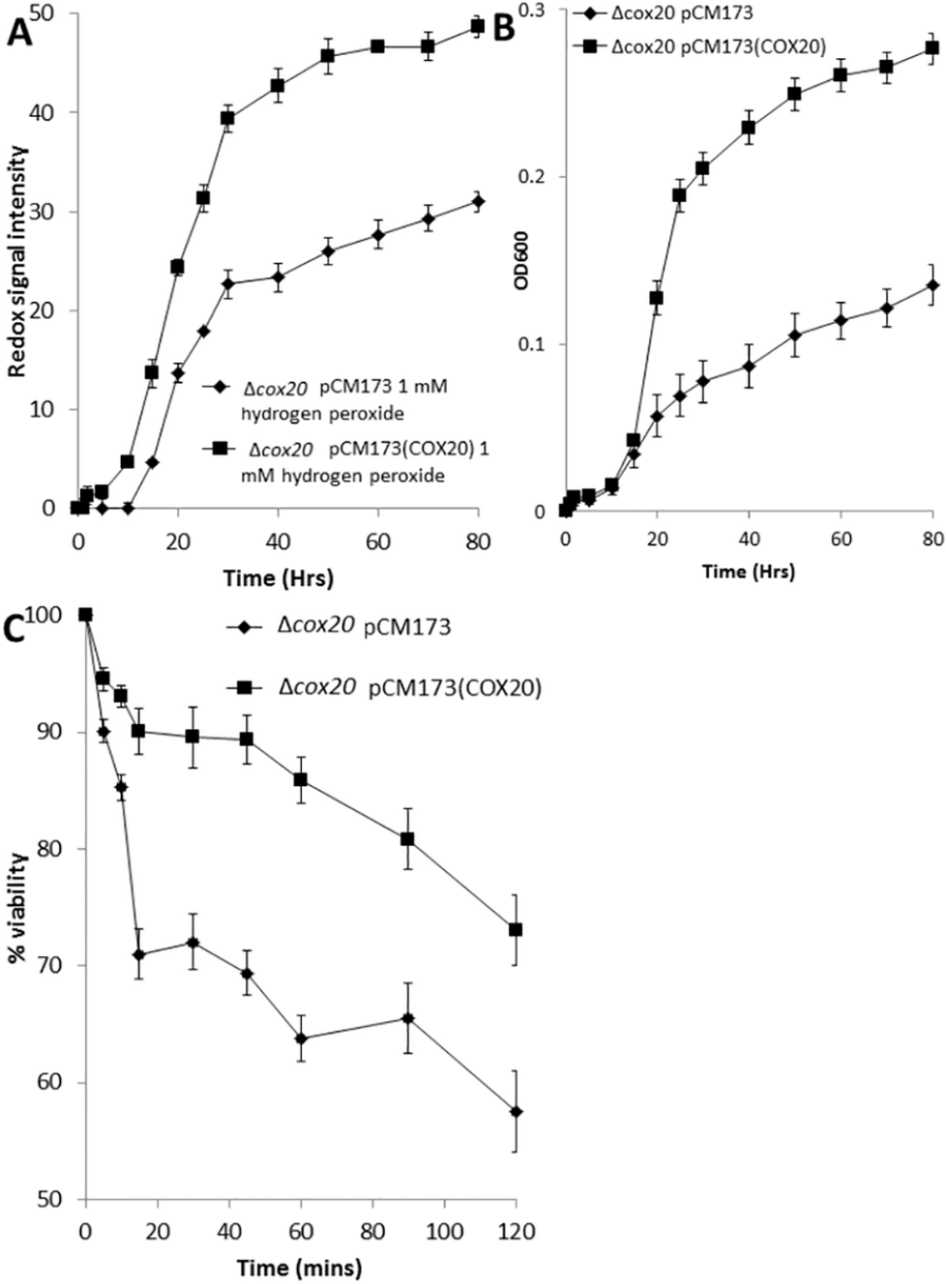
(A) Metabolic activity (redox signal intensity) of Δ*cox20* pCM173 or Δ*cox20* pCM173(*COX20*) in the presence of 1 mM hydrogen peroxide (B) Growth rates (OD600) for Δ*cox20* pCM173 or Δ*cox20* pCM173(*COX20*) in the presence of 1 mM hydrogen peroxide (C) Viability of Δ*cox20* pCM173 or Δ*cox20* pCM173(*COX20*) in the presence of 1 mM hydrogen peroxide measured over 120 mins. Results presented are a representative of triplicate values (Mean *+*/- SD n = 3).

Cytochrome C oxidases have been reported to be involved in cellular response to oxidative stress [17], we thus assessed whether overexpressing *COX20* had an effect on tolerance to hydrogen peroxide-induced oxidative stress. Assays revealed that overexpression of *COX20* increased metabolic output in the presence of 1 mM hydrogen peroxide when compared with assays using the empty vector containing yeast as a control (Fig 6A). We also observed an improvement in growth and viability in the presence of hydrogen peroxide for cells overexpressing *COX20* as compared to the empty vector controls (Fig 6B and 6C).

### Expressing *COX20* conferred advantages when utilising hydrolysates derived from wheat

**Fig 7:**
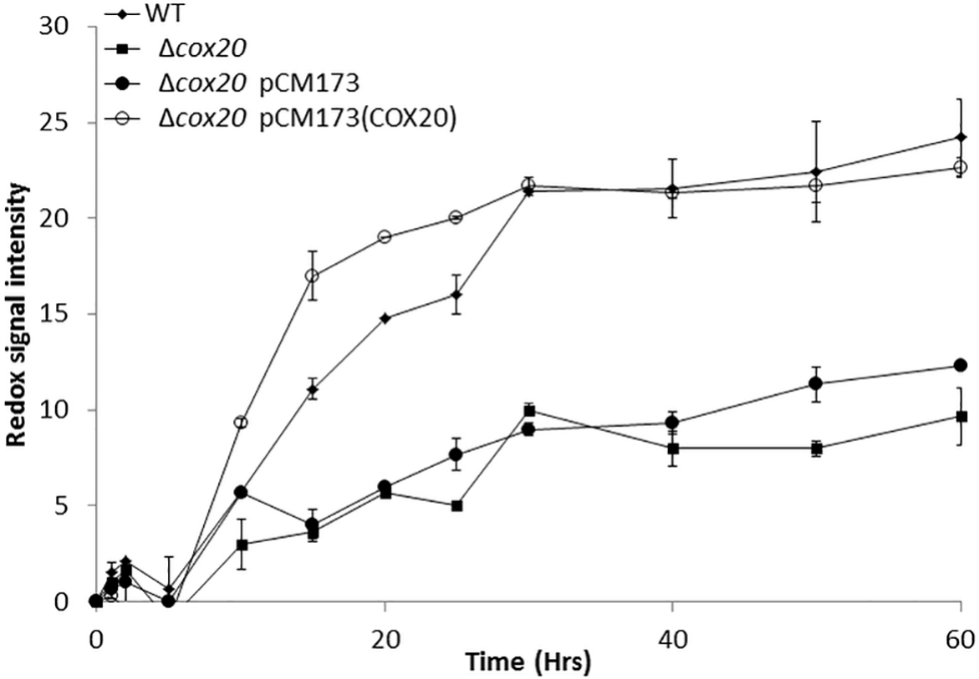
Metabolic activity (redox signal intensity) of WT, Δ*cox20*, Δ*cox20* pCM173, and pCM173(*COX20*) on hydrolysates derived from an acid pre-treatment of wheat. Results presented are a representative of triplicate values (Mean *+*/- SD n = 3).

We assessed for the importance of overexpressing *COX20* when utilising hydrolysates derived from the acid pre-treatment of wheat, hydrolysates with glucose added were assessed using a phenotypic microarray assay. It was observed that there was an improvement in metabolic output in a strain expressing *COX20* when compared with the empty vector control (Fig 7), however, when comparing a strain expressing *COX20* with the basal wild-type strain there was an initial improvement but that improvement was soon lost and the wild-type strain eventually outperforms the strain which overexpresses COX20 (Fig 7). These strains overexpress COX20 using a tryptophan auxotrophy as a selection requirement, these hydrolysates do contain tryptophan (data not shown) so the selection pressure for maintain strains containing the vector would be lost. This was proven in growth studies on SD-trp which after growth on hydrolysates there was no growth observed indicating a loss of vector in the system.

## Discussion

Presence of acetic acid is an unavoidable consequence of pre-treatment of lignocellulosic material into fermentable sugars [6, 20], which will inhibit yeast performance in fermentations. Previous work identified *COX20* up-regulated under acetic acid stress; however, the importance of the gene had not been fully examined. *COX20* encodes for a gene whose function is essential for the maturation of the mitochondrially encoded Cox2p, the pre-cursor of cytochrome C oxidase in yeast [15].

Presence of acetic acid (20–80 mM) has been shown to induce programmed cell death (PCD) [22], either involving mitochondria (intrinsic pathway) or a pathway involving cytosolic caspases called the extrinsic pathway [23, 24]. The toxicity of acetic acid is pH dependent, acetic acid in its undissociated form diffuses through the cell membrane and dissociation is dependent on cytosolic pH. Intracellular pH is maintained through ATPase transporter systems transporting protons across the cell membrane [25].

During fermentations, low concentrations of acetic acid (< 25 mM) stimulate ATP production, and under anaerobic conditions the rate of ethanol production increases compared with the rate in the unstressed control [26]. At higher concentrations (>25 mM) the stimulation in ATP production is overtaken by a lowering in cytosolic pH and an increase in ATPase activity.

Deletion of *COX20* had no discernible effect under control conditions; however, in the presence of acetic acid, a Δ*cox20* strain was sensitive when compared with the background strain. Overexpression of *COX20* increased tolerance to acetic acid, strains carrying a tetracycline-regulatable vector containing *COX20* showing no inhibition in the presence of 75 mM acetic acid. Placing *COX20* under a tetracycline-regulatable promoter allowed us to control tolerance to acetic acid as addition of tetracycline suppressed expression and returned a Δ*cox20* strain back to acetic acid sensitivity. As pre-treatment conditions become more stringent, concentrations of weak acids such as acetic, formic and levulinic acid also increase [6, 20] along with increasing concentrations of monomeric fermentable sugars [27] making a fermentation into bioethanol more viable [28]. The role of cytochrome C oxidases in acetic acid induced PCD has been well established, however this is the first report of overexpression of a gene product that could impact cytochrome C oxidase increasing tolerance to acetic acid during fermentation. Overexpression of *COX20* allowed conversion of glucose into ethanol at close to the theoretical maximum yield in the presence of 75 mM acetic acid.

Mitochondria are the major site within the cell for the generation of ROS, and incorrect assembly of cytochrome C oxidase complexes has been associated with accumulation of ROS within the yeast cell [17]. We assessed the impact of overexpressing *COX20* on cells under hydrogen peroxide-induced oxidative stress and observed an improved metabolic output, growth and viability in those strains when compared with empty vector control.

Acetic acid tolerance in *Saccharomyces* spp has been defined previously [29, 30] with considerable variation observed within the species. Determining if cytochrome C oxidase activity in these strains correlated with tolerance would be of interest.

## Conclusion

Results here revealed that use of tetracycline-regulated system in a fermentation correlated expression of a desired gene with a phenotype; this work follows up a similar study from our previous research group [31]. Cytochrome C oxidase activity under acetic acid stress has been highlighted previously as pre-cursors for programmed cell death in yeast; however expression of *COX20* correlating with acetic acid or oxidative tolerance has not been shown previously. *COX20* expression correlated with a tolerant phenotype to acetic acid and this tolerance was reverted upon addition of tetracycline into the assay.

## Supporting Information

S1 Fig Phenotypic microarray analysis of Δ*cox20* pCM173, Δ*cox20* pCM173(*COX20*) under A) 100 mM acetic acid and (B) 20 mM formic acid. Results presented are a representative of triplicate values (Mean *+*/- SD n = 3).
(PPTX)

S2 Fig (A) Fermentation rates (weight loss) for Δ*cox20* pCM173 and Δ*cox20* pCM173(*COX20*) under control conditions (4% glucose), (B) Fermentation rates (weight loss) for Δ*cox20* pCM173 and Δ*cox20* pCM173(*COX20*) under 75 mM acetic acid, (C) fermentation rates for Δ*cox20* pCM173(*COX20*) under absence and presence of 75 mM acetic acid. Results presented are a representative of triplicate values (Mean *+*/- SD n = 3).
(TIF)

S3 Fig Acetic acid concentrations (mM) dring a fermentation for Δ*cox20* pCM173, Δ*cox20* pCM173(*COX20*) under (A) absence or (B) presence of 75 mM acetic acid. Results presented are a representative of triplicate values (Mean *+*/- SD n = 3).
(TIF)
