# Supplementary figures and images for: Expression of Mitochondrial Cytochrome C Oxidase Chaperone Gene (COX20) Improves Tolerance to Weak Acid and Oxidative Stress during Yeast Fermentation
Source: PLoS One. 2015 Oct 1;10(10):e0139129. doi: 10.1371/journal.pone.0139129 (PMC4591339; doi:10.1371/journal.pone.0139129)

## Slide 1
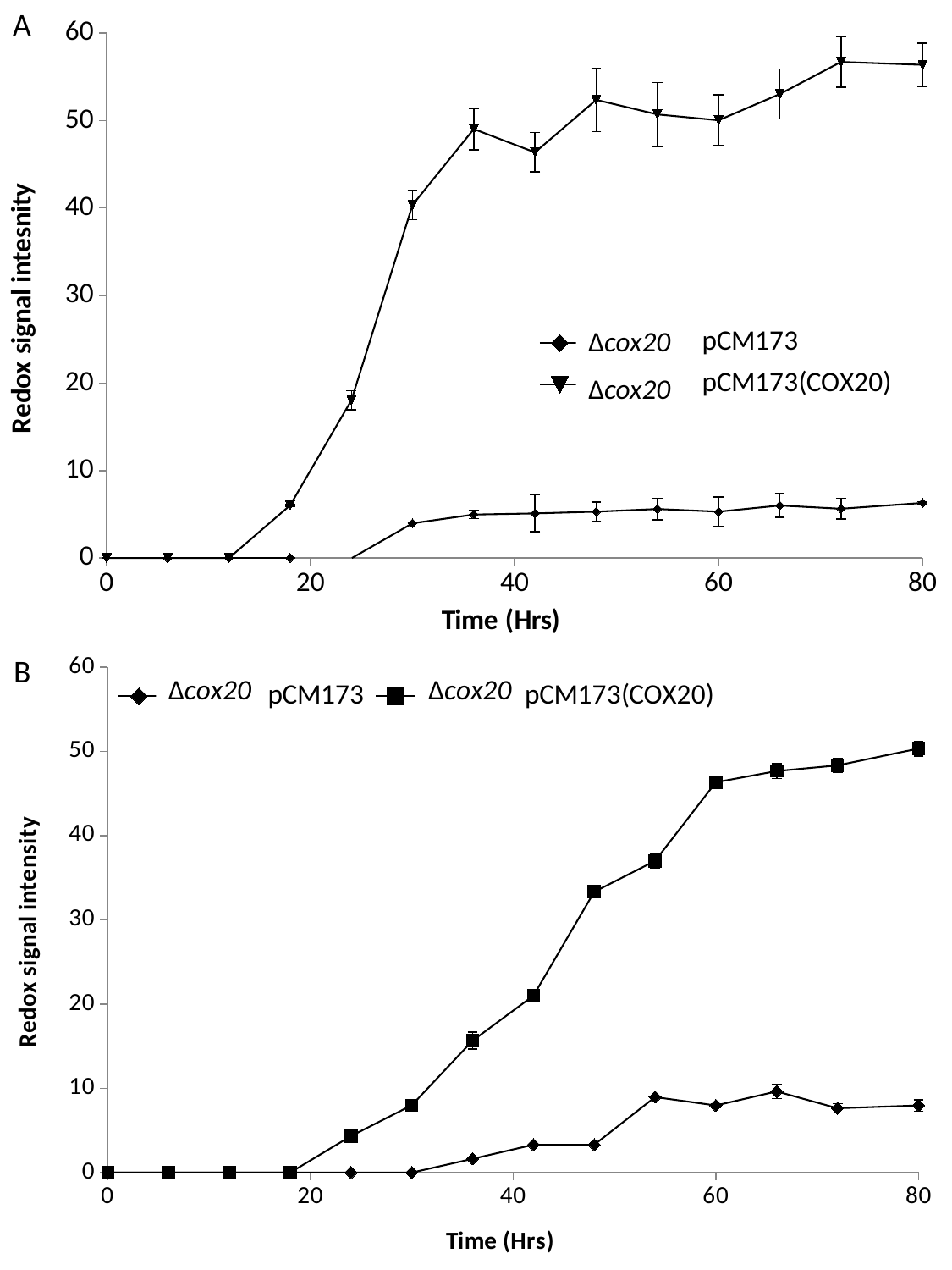

### Chart
| Category | Δcox20 pCM173 | Δcox20 pCM173(COX20) |
|---|---|---|A
Δcox20
Δcox20
### Chart
| Category | Δcox20 pCM173 | Δcox20 pCM173(COX20) |
|---|---|---|B

Supplement: S1 Fig — Results presented are a representative of triplicate values (Mean +/- SD n = 3). (PPTX) [file pone.0139129.s001.pptx]

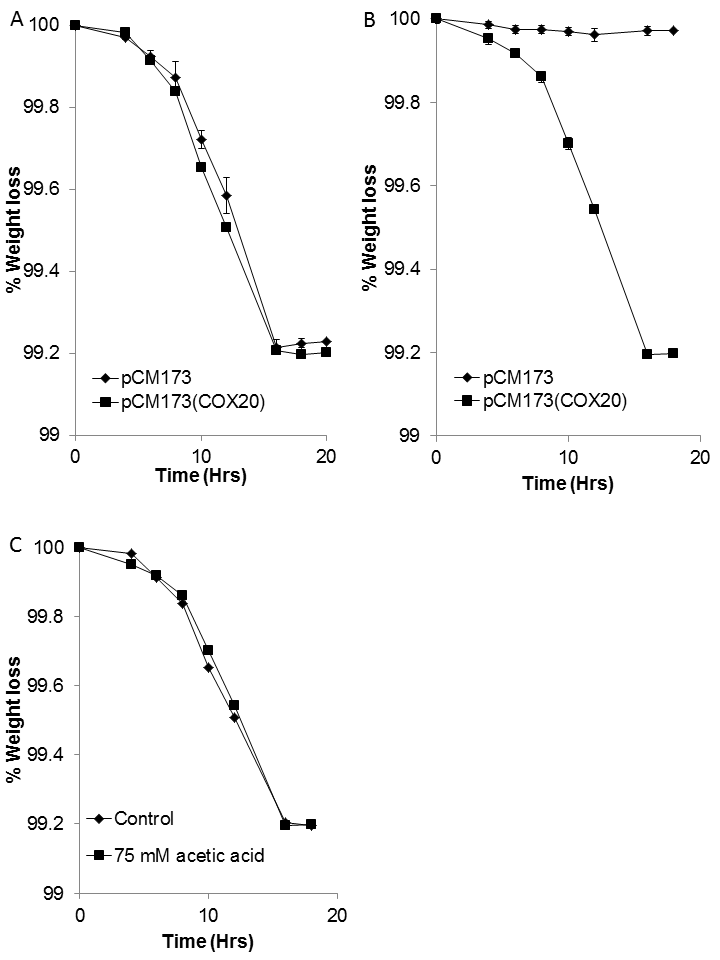

Supplement: S2 Fig — Results presented are a representative of triplicate values (Mean +/- SD n = 3). (TIF) [file pone.0139129.s002.tif]

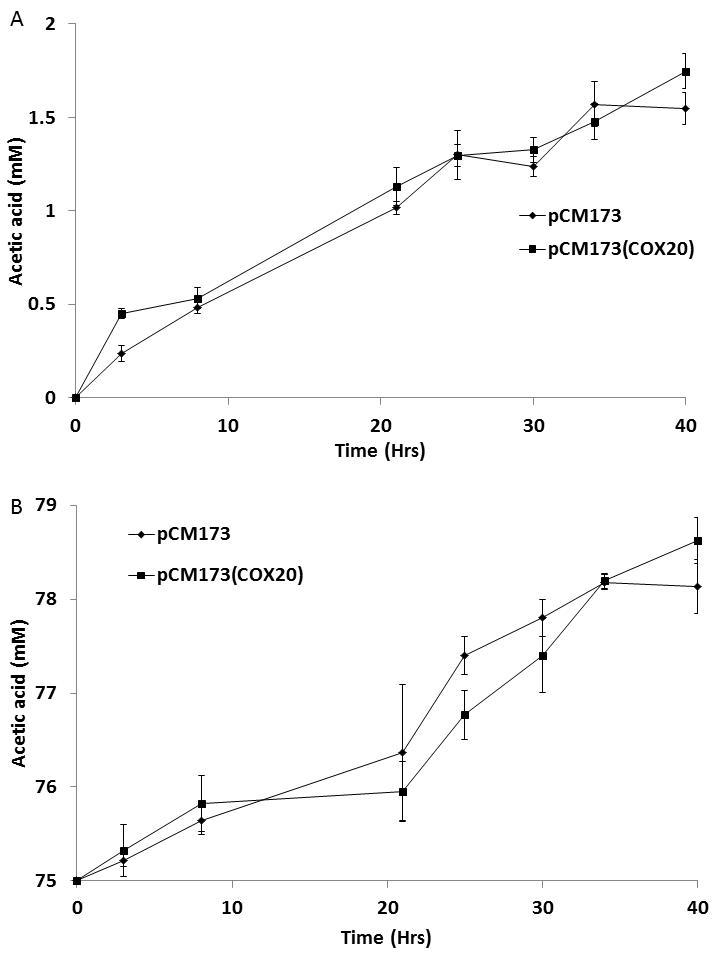

Supplement: S3 Fig — Results presented are a representative of triplicate values (Mean +/- SD n = 3). (TIF) [file pone.0139129.s003.tif]
